# A New Stemness-Related Prognostic Model for Predicting the Prognosis in Pancreatic Ductal Adenocarcinoma

**DOI:** 10.1155/2021/6669570

**Published:** 2021-10-11

**Authors:** Xiao-Yan Huang, Wen-Tao Qin, Qi-Sheng Su, Cheng-Cheng Qiu, Ruo-Chuan Liu, Shan-Shan Xie, Yang Hu, Shang-Yong Zhu

**Affiliations:** ^1^Department of Medical Ultrasound, First Affiliated Hospital of Guangxi Medical University, 6 Shuangyong Rd, 530021 Nanning, Guangxi, China; ^2^Department of Bone and Joint Surgery, First Affiliated Hospital of Guangxi Medical University, 6 Shuangyong Rd, 530021 Nanning, Guangxi, China; ^3^Department of Clinical Laboratory, First Affiliated Hospital of Guangxi Medical University, 6 Shuangyong Rd, 530021 Nanning, Guangxi, China

## Abstract

**Objective:**

This study is aimed at identifying stemness-related genes in pancreatic ductal adenocarcinoma (PDAC).

**Methods:**

The RNA-seq data of PADC patients were downloaded from The Cancer Genome Atlas (TCGA) and Genotype-Tissue Expression (GTEx) databases. The mRNA expression-based stemness index (mRNAsi) and epigenetically regulated mRNAsi (EREG-mRNAsi) of PADC patients were evaluated. The mRNAsi-related gene sets in PADC were identified by weighted gene coexpression network analysis (WGCNA). The key genes were further analyzed using functional enrichment analysis. The Kaplan-Meier survival analysis and the Cox proportional hazards model were used to evaluate the prognostic value of the key genes. Prognostic hub genes were used to establish nomograms. The receiver operating characteristic (ROC) curves, concordance index (*C*-index), and calibration curves were used to assess the discrimination and accuracy of the nomogram. Finally, these results were validated in the Gene Expression Omnibus (GEO) database.

**Results:**

A total of 36 key genes related to mRNAsi were identified by WGCNA. A prognostic gene signature compromising seven genes (TPX2, ZWINT, UBE2C, CCNB2, CDK1, BUB1, and BIRC5) was established to predict the overall survival (OS) of PADC patients. The Cox regression analysis revealed that the risk score was an independent prognostic factor for PADC. Patients were then divided into the high-risk and low-risk groups. The ROC curves, *C*-index, and calibration curves indicated good performance of the prognostic signature in the TCGA and GEO datasets. Moreover, the nomogram incorporating clinical parameters showed better sensitivity and specificity for predicting the OS of PADC patients.

**Conclusion:**

The stemness-related prognostic model successfully predicted the OS of PADC patients and could be used for the treatment of PADC.

## 1. Introduction

Pancreatic ductal adenocarcinoma (PDAC) is the most prevalent type of pancreatic neoplasm, accounting for more than 90% of the total number of pancreatic tumors. The 5-year survival rate of PDAC is less than 10%. Moreover, PDAC is expected to become the second leading cause of cancer-related death by 2030 [1, 2]. Surgical resection represents the only chance for cure and the advances in adjuvant chemotherapy have improved the long-term outcomes of PDAC patients [3]. Early diagnosis and effective intervention are the major factors for favorable outcomes in PADC. However, current treatments often cause trauma, which impairs the quality of life of patients. Molecular targeted therapy, which inhibits cancer growth, progression, and metastasis by targeting specific molecular biomarkers, has emerged as a promising treatment strategy with better efficacy and fewer trauma-related complications [4]. However, the identification of molecular biomarkers remains challenging due to insufficient understanding of the pathogenic mechanisms of PDAC.

Cancer stem cells (CSCs) have been shown to promote tumor recurrence, metastasis, and drug resistance owing to their self-renewal ability, proliferation capacity, and multilineage differentiation potential [5]. Emerging evidence has indicated that the stemness of CSCs is associated with high intratumoral heterogeneity in most types of cancers, including PADC [6, 7]. Intratumoral heterogeneity, which includes phenotypic diversity (e.g., cell surface markers and epigenetic abnormality), has been reported to drive disease progression and cause treatment failure. These markers and gene mutation types are often used for pathological classification and clinical treatment of tumors [8]. Pancreatic CSCs, first described in 2007 [9], account for less than 1% of all pancreatic cancer cells [10]. They are responsible for the development, metastasis, and chemoresistance of PDAC. The activation of CSC-related biomarkers and signaling pathways, including CD133, CD24, CD44, MYC, WNT/*β*-catenin, and Notch, has been reported in PDAC [11]. Although previous studies have explored the unlimited self-renewal capacity of pancreatic CSCs and their roles in tumorigenesis and chemoresistance, investigations on the molecular mechanisms of pancreatic CSCs are still warranted. In this study, we aimed to uncover the heterogeneity of PADC from the perspective of the stemness features of CSCs.

The stemness properties of CSCs are mainly characterized by mRNA expression-based stemness index (mRNAsi), such as epigenetically regulated mRNAsi (EREG-mRNAsi) [5]. The mRNAsi score has been used to identify new CSC markers and to indicate carcinogenic dedifferentiation [12]. Previous studies have reported that the mRNAsi score is associated with the stemness features of CSCs and can be calculated by the one-class logistic regression (OCLR) machine learning algorithm, suggesting a significant correlation between mRNAsi and the prognosis of PADC [5]. However, genes related to the stemness features of CSCs have not been fully identified and the biological functions of these genes remain largely unknown. The analysis of differentially expressed genes (DEGs) has been used to identify key genes related to tumorigenesis, but it is unable to elaborate the connections between those genes. The gene network analysis is a tool used to investigate the complex process of tumorigenesis [8]. The weighted gene coexpression network analysis (WGCNA) has been used to explore coexpression modules associated with the clinical characteristics of cancer patients, including mRNAsi [13, 14].

In the current study, WGCNA was used to identify stemness-related modules and key genes. The functions of these genes during the development of PADC were explored using Gene Ontology (GO) analysis, Kyoto Encyclopedia of Genes and Genomes (KEGG) analysis, and Gene Set Enrichment Analysis (GSEA). A novel prognostic model comprising seven genes (i.e., TPX2, ZWINT, UBE2C, CCNB2, CDK1, BUB1, and BIRC5) was then constructed and validated using the TCGA and GEO databases. Finally, prognostic genes were used to establish nomograms. The new model and nomogram might be a powerful tool for the prediction of the prognosis of PADC patients.

## 2. Material and Methods

### 2.1. Dataset Sources

Patients who met the following criteria were selected from the TCGA PADC cohort: (1) patients with histologically confirmed primary PADC, (2) patients with RNA-seq data, and (3) patients with complete clinicopathological data, such as age, gender, TNM stage, grade, and overall survival (OS). The corresponding RNA-seq data and clinicopathological information of PADC patients were collected from the TCGA in the GDC database (https://portal.gdc.cancer.gov/). The expression of genes in normal tissues was obtained from the GTEx database. All data were available on September 9^th^, 2020. The RNA-seq data, including 169 normal samples and 142 tumor samples, were merged into a matrix file using a merge script in Perl language (https://www.perl.org/).

### 2.2. mRNAsi in PADC and Its Clinical Significance

The mRNAsi used for assessing the degree of oncogenic dedifferentiation was obtained from previous studies [5]. The mRNAsi score of PADC samples was calculated using the PADC dataset in the TCGA database by OCLR. The gene expression-based stemness index ranges from low (zero) to high (one) stemness. Significant differences in mRNAsi between tumors and nontumors were determined by the Wilcoxon test. To evaluate the prognostic value of the mRNAsi score, an OS analysis according to the mRNAsi score was performed using the “survival” package in the R software (v3.6.1, https://CRAN.R-project.org/package=survival). The Kaplan-Meier (K-M) analysis of samples with high or low mRNAsi scores was carried out. The Wilcoxon test was then performed to investigate the correlation between the mRNAsi score and patient's age.

### 2.3. DEGs in PADC

The Wilcoxon test was used to identify DEGs between PADC and normal tissue samples. A false discovery rate (FDR) of <0.05 and a ∣log2 fold change (FC) |  of ≥ 1 were set as the cut-off criteria for defining DEGs.

### 2.4. WGCNA Construction and Module Preservation

An R package “WGCNA” was used to investigate the correlations among genes by constructing modules. The modules were obtained by clustering genes with similar expression patterns [14]. A total of 1750 DEGs with high precision and accuracy were selected for subsequent analysis. To achieve high-scale independence and mean connectivity, the soft-thresholding power was calculated using a gradient test ranging from 1 to 20 and determined by the pickSoftThreshold function. Then, the topological overlap matrix (TOM) was calculated based on the corresponding soft-thresholding power. The TOM was used to identify the modules of highly coexpressed genes and to make the networks less sensitive to spurious connections. Genes with high absolute correlations were further clustered into the same module. The modules were defined by cutting the clustering tree into branches using a dynamic tree cutting algorithm and then assigned to different colors for visualization. The module dendrograms were constructed using hierarchical clustering analysis based on TOM-based dissimilarity. To avoid oversplitting, correlated modules (*r* < 0.25) were merged, and each module was labeled with the corresponding color [15].

### 2.5. Identification of Modules Associated with Clinical Characteristics

Module eigengenes (MEs) are considered the major components in the principal component analysis for each gene module. The expression patterns of all genes can be summarized as a single expression profile within a given module [16]. The gene significance (GS) was calculated to demonstrate the correlation between genes and clinical characteristics. The module significance (MS), referring to the average GS of all genes in the module, was used to determine the correlation between modules and the characteristics of samples. The module with a higher correlation with the MS was identified for further analysis.

### 2.6. Identification of Key Genes in the Red Module

The key genes in the coexpressed network were highly interconnected with the nodes in a module and were used to explore the biological functions of identified dysregulated genes [17]. The module membership (MM) was defined as the correlation of the gene expression profile with MEs. The key genes in the module were defined as cor. GS > 0.5 and cor. gene MM > 0.8. The R package “corrplot” was used to calculate Pearson's correlation coefficient among these genes.

### 2.7. Functional and Pathway Enrichment Analyses

To investigate the biological functions of the key genes, GO and KEGG analyses were performed using the R package “clusterProfiler” [18, 19]. An FDR of ≤0.05 was considered statistically significant. A protein-protein interaction (PPI) network of the key genes in key modules was constructed using the STRING (https://www.string-db.org/) [20]. Hub nodes were identified when the combined score was ≥0.8, and the connectivity degree was ≥20.

### 2.8. Establishment of a Prognostic Model

The K-M survival analysis and the stratified Cox proportional hazards analysis were used to evaluate the prognostic value of the key genes. Then, the risk score of each prognostic gene was calculated based on the sum of Si (expression level of the key gene) and *β* (corresponding coefficient) generated from the Cox model. PADC patients were divided into the high-risk and low-risk subgroups according to the median risk score, and their OS was analyzed using the K-M analysis. According to the method proposed by Blanche et al. [21], the predictive accuracy of each prognostic signature was accessed by calculating Uno's inverse-probability of censoring weighting estimation of the time-dependent receiver-operator characteristic (ROC) area under the curve (AUC) values (time spanning from 6 to 24 months) using the “timeROC” package (version 0.3). Finally, the expression of the signature genes was visualized in the heatmap using the R package “pheatmap.”

### 2.9. Prognostic Value of the Prognostic Model

The risk scores and clinicopathological parameters, including age, gender, TNM stage, grade, OS, postoperative radiotherapy (postoperative_tx_tx), radiotherapy, and alcohol consumption history, were included in the univariate and multivariate Cox regression analyses. Based on the results of the multivariate Cox regression analysis, a nomogram was established using the “rms” package (version 5.1.2) [22]. The nanogram was used to predict the 6-, 12-, and 24-month OS of PADC patients in the TCGA dataset. Subsequently, a time-dependent ROC curve was plotted to assess the sensibility and specificity of the nomogram using the R package “timeROC.” The AUC was also calculated. The predictive accuracy of the nomogram was assessed by the nomogram calibration curve and Harrell's *C*-index. The calibration curve was plotted to determine the consistency between the predicted and observed OS. The *C*-index was calculated using a bootstrap method with 1000 resamples to assess the discrimination ability of the nomogram.

### 2.10. Validation of the Prognostic Signature in the GEO Database

To minimize bias caused by small sample size, the prognostic capacity of the model was validated using the GSE62452 dataset in the GEO database (http://www.ncbi.nlm.nih.gov/geo/). The optimal risk score cut-off value for the GEO dataset was the same as that for the primary TCGA cohort. The survival curves of the low-risk and high-risk subgroups were plotted using the K-M method. The predictive accuracy of the prognostic model was evaluated using ROC curves. The performance of the diagnostic nomogram was assessed by time-dependent ROC curves and calibration curves.

### 2.11. Gene Set Enrichment Analysis (GSEA)

To identify the potential function of the prognostic model, GSEA (https://www.gsea-msigdb.org/gsea/index.jsp) was performed using the GSEA software (v4.0.3). GSEA determines whether a priori defined set of genes shows statistically significant, concordant differences between two biological states [23]. Based on the molecular signature database (v. 6.2), C2 (curated gene sets), C5 (GO gene sets), and C6 (oncogenic signatures) were analyzed to identify KEGG pathways, biological processes (BP), cellular components (CC), molecular functions (MF), and dysregulated oncogenic signatures. The samples in TCGA were divided into two groups ( the high-risk score group vs. low-risk score group) according to the median expression value of each gene. An enriched gene set with a nominal *p* value of <0.05, a |NES| of >1, and a FDR *q* value of <0.25 was considered statistically significant.

### 2.12. Statistical Analysis

Statistical analysis was performed using the R software (v3.6.1). In the K-M survival analysis, *p* value and hazard ratio (HR) with 95% confidence interval (CI) were generated by the log-rank test and univariate Cox proportional hazards regression analysis. Pearson's Chi-square test was performed to assess the significance between groups. The results of multivariate Cox regression analysis were visualized by the nomogram. *C*-index, time-dependent ROC curves, and calibration curves were used to evaluate the nomogram. A *p* value of <0.05 was considered statistically significant.

## 3. Results

### 3.1. Data Processing and Survival Analysis

**Figure 1:**
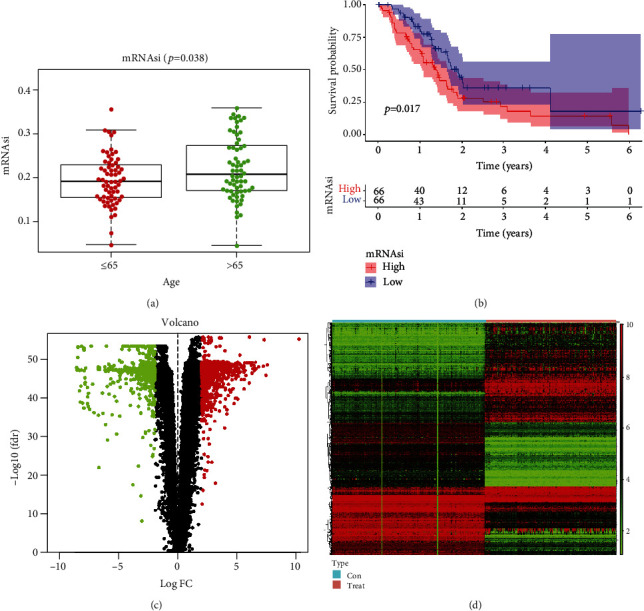
The differentially expressed of mRNAsi in different PADC subtypes. (a) Boxplots of mRNAsi in individual samples with age (over 65 or not). (b) Kaplan-Meier survival analysis showed that there were significantly differences between high mRNAsi and low mRNAsi groups in PADC. (c, d) The volcano and heatmap showed the upregulated (red) and downregulated (green) genes in PADC.

CSC-related RNA-seq data were obtained from the TCGA PADC dataset, including 142 PADC samples. The data of 169 normal samples were collected from the TCGA and GTEx databases. The gene expression-based stemness index for PADC was extracted by OCLR [5]. The mRNAsi indicates the degree of similarity between tumor cells and stem cells. The EGER-mRNAsi is an index that describes the epigenetic regulation of CSC-related genes. As shown in Figure 1(a), there was a significant difference in age between those younger than 65 and those older than 65 years. The survival analysis showed that the level of mRNAsi was significantly different from the survival time of PADC patients (Figure 1(b), adjusted *p* < 0.0017), further suggesting that the stemness features of CSCs were related to the survival outcomes of PADC patients.

### 3.2. Identification of DEGs in PADC

A total of 1750 DEGs were identified using the Wilcoxon test, including 845 downregulated genes and 905 upregulated genes. The expression of these genes was visualized using the R package “beeswarm.” The volcano plot and heatmap show the DEGs between PADC and normal tissues (Figure 1(c)).

### 3.3. Construction of 14 Co-Expression Modules by WGCNA

**Figure 2:**
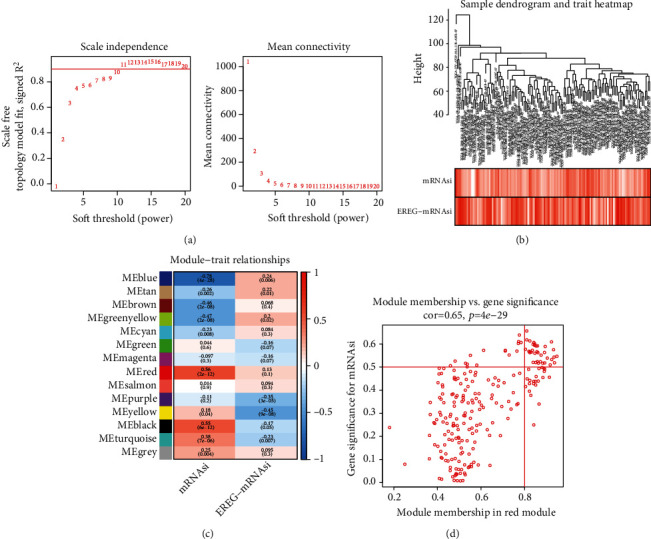
Identification of the hub modules in TCGA PADC cohorts. (a, A) The left scale free index indicates the correlation scale free fit index (*y*-axis) and soft-thresholding power (*x*-axis). (B) The average connectivity (*y*-axis) corresponding to different soft-thresholding power (*x*-axis). The approximate scale-free topology can be attained the soft-thresholding power of 11. (b) The red represents the mRNAsi scores; the darker the color, the higher the value. (c) Heatmap of the correlation between module eigengenes and clinical traits. The clinical traits include mRNAsi and EGER-mRNAsi. Each cell contains the corresponding correlations and *p* values. (d) The red module represents the highest positive correlation with stemness characteristics of cancer stem cells (CSCs).

**Figure 3:**
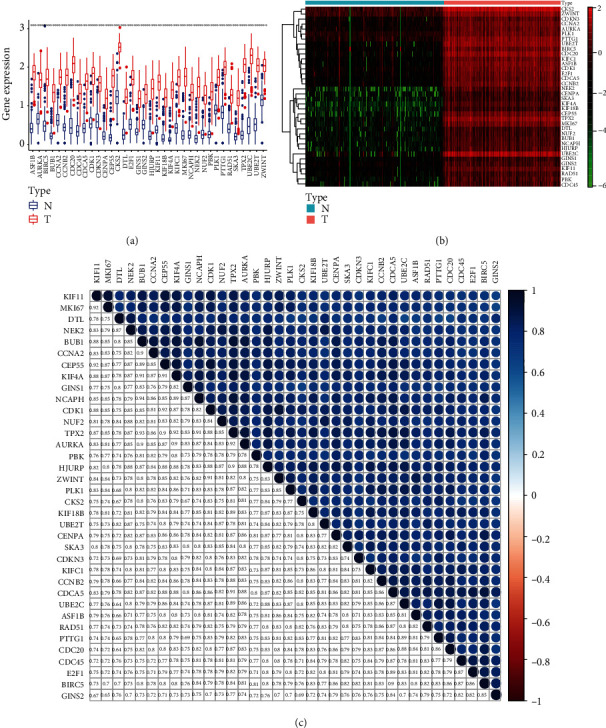
The differential expression of 36 the stem cell signature genes in PADC. (a, b) The differential expression of 44 hub genes in red module. (c) The interrelationship of the upregulated genes at transcriptional level.

Based on DEGs, WGCNA was performed to identify stemness-related modules. A power of *β* = 11 (scale-free *R*^2^ = 0.95) was set as soft threshold (power) to ensure a scale-free network (Figure 2(a)). Then, we established a sample dendrogram and a trait heatmap of mRNAsi and EGER-mRNAsi (Figure 2(b)). According to the cluster analysis of PADC, there were 14 different modules (module size ≥ 50, cut height ≥ 0.25) in the network (blue, tan, brown, green-yellow, cyan, green, magenta, red, purple, yellow, black, turquoise, and grey) (Figure 2(c)). Three modules were related to the mRNAsi. The red module showed the highest positive correlation with the mRNAsi (Figure 2(c), r = 0.56, *p* = 2*e* − 12). The blue module exhibited the most significant negative correlation with the mRNAsi (Figure 3(c), r = −0.78, p = 4e − 28). Thus, the red module was used to further explore the hub genes (Figure 2(d)).

### 3.4. Analysis of the Key Gene in the Red Module

Genes in the same module exhibit common expression patterns. In the red module, 36 key genes out of 1750 DEGs were related to the mRNAsi. We further compared the DEGs with the minimum *p* value (p < 0.01) in PADC and normal tissues and found that all of them were upregulated in tumor tissues (Figure 3(a)). Meanwhile, we generated a heatmap using the R package “ggpubr” to display the DEGs between tumor and normal tissues (Figure 3(b)). Moreover, the relationships among key genes were visualized in a heatmap (Figure 3(c), p < 0.01).

### 3.5. Functional Enrichment and PPI Network Analyses

**Figure 4:**
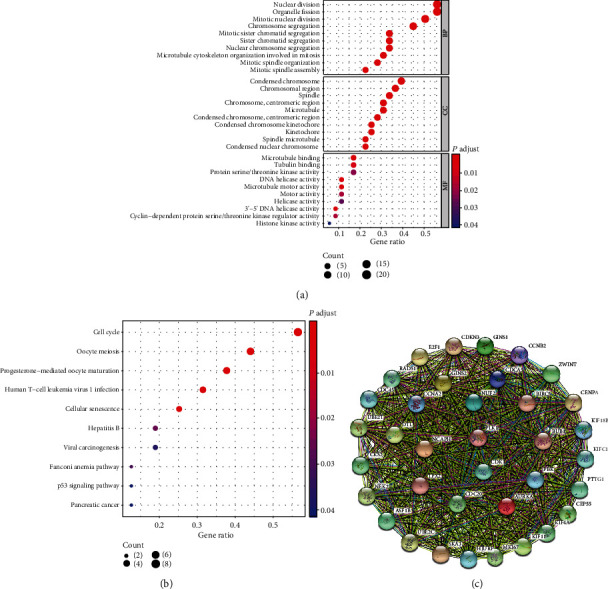
Functional enrichment analysis in the red module. (a, b) Functional analysis for 36 stemness-related genes of CSCs by GO and KEGG analyses in PADC. (c) The protein-protein interaction network of 36 hub genes in PADC.

To further explore the biological functions of 36 key genes in PADC, GO enrichment and KEGG pathway analyses were performed. The functions of these genes were mainly enriched in 30 pathways, including BP (biological process), CC (cellular component), and MF (molecular function) (Figure 4(a)). BP was found to be relevant to mitotic nuclear division. CC was related to the spindle. MF includes microtubule binding. As shown in Figure 4(b), KEGG pathway analysis indicated that hub genes were enriched in the cell cycle signaling pathway. The signaling pathway with an FDR-corrected *p* value of <0.01 was considered significant. Furthermore, a PPI network of key genes was constructed to identify gene-gene interaction (Figure 4(c)).

### 3.6. Identification and Evaluation of the Stemness-Related Prognostic Model

**Figure 5:**
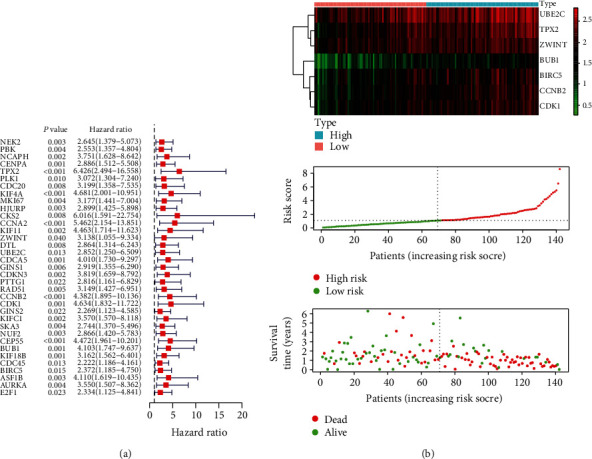
(a, b) Risk score analysis, survival status, and survival time between the two risk groups and expression distribution of the seven-gene signature in the TCGA dataset.

To evaluate the prognostic value of stemness-related genes, a univariate Cox regression analysis was performed. The results showed that 36 key genes were associated with the OS of PADC patients. Among them, 35 OS-related genes were identified as risk factors (Figure 5(a)). Next, a prognostic model comprising seven genes was developed based on the red module using the stepwise multivariate Cox regression analysis. The seven genes were TPX2, ZWINT, UBE2C, CCNB2, CDK1, BUB1, and BIRC5. The risk score was calculated based on the Cox coefficients of these genes:

(1) $$\begin{array}{c} \text{Risk score}=5.05684*\text{Exp}\left(\text{TPX}2\right)-4.06061*\text{Exp}\left(\text{ZWINT}\right)-3.02086*\text{Exp}\left(\text{UBE}2\text{C}\right)+3.3769*\text{Exp}\left(\text{CCNB}2\right)+3.716557*\text{Exp}\left(\text{CDK}1\right)-2.59406*\text{Exp}\left(\text{BUB}1\right)-1.12413*\text{Exp}\left(\text{BIRC}5\right). \end{array}$$

**Figure 6:**
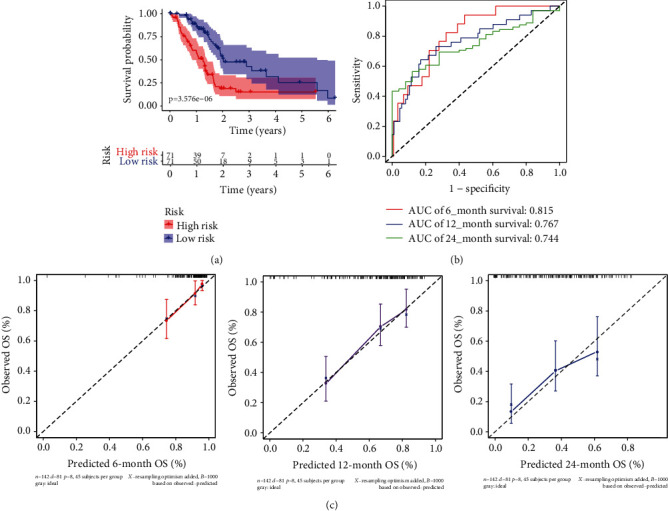
Establishment and verification of the prognostic model. (a) The survival time of patients in the high-risk group was significantly longer than that of patients in the low-risk group (*p* < 0.0001). (b) The AUC of the ROC and risk score models for predicting 6-, 12-, and 24-month survival showed good accuracy. (c) Calibration plot for predicting probabilities of 6-, 12-, and 24-month OS of primary PADC patients in the TCGA database.

Then, patients were divided into two groups, the high-risk group (risk score ≥ 1.103) and the low-risk group (risk score < 1.103). The survival of all PADC patients and the heatmap of seven prognostic genes are shown in Figure 5(b). The K-M curves revealed that the high-risk group had a significantly worse prognosis compared to the low-risk group (*p* < 0.0001) (Figure 6(a)). Furthermore, in the validation dataset, the 6-, 12-, and 24-month AUCs of the time-dependent ROC curves were 0.815, 0.767, and 0.744, respectively (Figure 6(b)), suggesting high efficiency of the seven-gene prognostic signature in predicting the OS of PADC patients. The *C*-index of the risk score was 0.718 (95% CI; 0.148–1.289). The calibration curves of the nomogram for 6-, 12-, and 24-month survival probabilities are shown in Figure 6(c).

### 3.7. Construction and Validation of a Prognostic Nomogram

**Figure 7:**
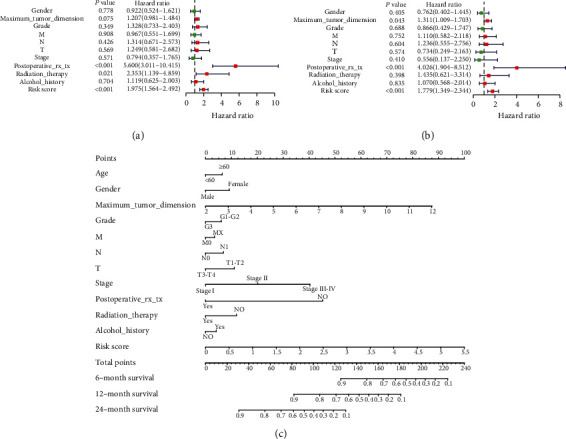
Analysis of clinicopathological information with OS. (a) Univariate and (b) multivariate analyses of clinicopathological information with OS. (c) A nomogram including risk score and other clinical features for predicting 6, 12, and 24 months of OS in PADC.

A prognostic nomogram for predicting the OS of PADC patients was developed based on the clinical data of 311 patients from the TCGA database. The parameters of the nomogram included risk score, gender, tumor size, TNM stage, grade, postoperative_tx_tx, radiotherapy, and alcohol consumption history. The forest plots of the univariate and multivariate Cox regression analyses based on 8 clinicopathological characteristics were used to evaluate the independent prognostic value of the signature (Figures 7(a) and 7(b)). The univariate Cox regression analysis showed that the risk group (HR = 1.975, *p* < 0.001), postoperative_rx_tx (HR = 5.600, *p* < 0.001), and radiation therapy (HR = 0.398, *p* = 1.435) were protective factors. Furthermore, the multivariable Cox regression analysis revealed that the risk group (HR = 1.779, *p* < 0.001), tumor size (HR = 1.311, *p* < 0.043), grade (HR = 0.866, *p* < 0.688), and postoperative_rx_tx (HR = 4.026, *p* < 0.001) were statistically significant. The *C*-index of the nomogram was 0.77373974 (0.6983633-0.8491161). The ROC curves demonstrated that risk score (0.706), gender (0.566), tumor size (0.618), grade (0.597), T stage (0.501), N stage (0.530), M stage (0.507), stage (0.539), postoperative_rx_tx (0.755), radiotherapy (0.580), and alcohol consumption history (0.572) had a high predictability (Figure 7(c)).

### 3.8. Validation of the Seven-Gene Prognostic Model

**Figure 8:**
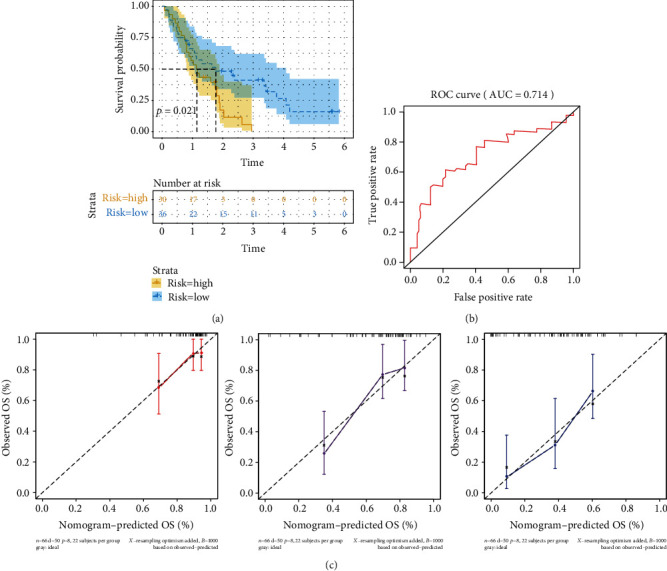
Validation of the seven-gene prognostic signature for PADC patients in GEO database. (a) Kaplan-Meier curves of OS for patients in the low- and high-risk groups (*p* < 0.0001). (b) ROC curves for 24 months. (c) Calibration curves for 6-, 12-, and 24-month OS of PADC patients in GEO datasets.

Next, we evaluated the predictive power of the prognostic gene signature in the PADC cohort from the GEO database (GSE62452). Similar procedures were carried out to assess the performance of the stemness index-associated signature. Patients were divided into the low-risk and high-risk groups according to their risk scores. Then, the OS of the two groups was compared. As shown in Figure 8(a), the high-risk cohort had a significantly poorer prognosis compared with the low-risk cohort (*p* < 0.021). Also, the time-dependent ROC showed that the AUC for 24-month OS was 0.714 (Figure 8(b)). The *C*-index for external validation set was 0.698 (95% CI, 0.625–0.770). The calibration curves of the nomogram predicting the 6-, 12-, and 24-month OS are shown in Figure 8(c). These results were consistent with those obtained in the TCGA database, suggesting the potential use of the seven-gene prognostic model in the diagnosis and treatment of PADC.

### 3.9. Gene Set Enrichment Analysis (GSEA)

**Figure 9:**
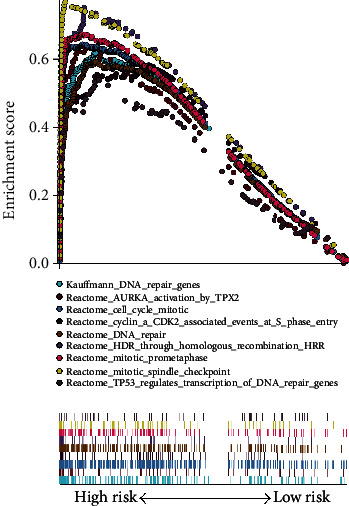
Gene set enrichment analysis between the high- and low- risk group in TCGA PADC cohort.

To further explore the functions of the seven genes in PADC, GSEA was performed. The results showed that these genes were associated with cell cycle mitotic, homologous recombination, mitotic nuclear division, oocyte meiosis, DNA replication, and the p53 signaling pathway (Figure 9).

## 4. Discussion

PDAC is a heterogeneous malignancy with high morbidity and mortality. CSCs, a small proportion of cells within the tumor, possess unlimited proliferative potential and share similar properties with cancer cells. Previous studies have suggested that the regulatory mechanisms underlying the self-renewal of stem cells and tumor cells are similar. Moreover, tumor cells may be derived from normal stem cells [24–26]. CSCs were first identified in a leukemia model, in which CD34^+^CD38^−^ leukemic cells showed the characteristics of bone marrow hematopoietic stem cells [27, 28]. Solid tumor CSCs (CD44^+^CD24^−/low^Lineage^−^ cells) have been found in breast, ovarian, prostate, colon, pancreatic, liver, and lung cancers, suggesting that CD34^+^ and CD44^+^ are typical CSC markers [29, 30]. In addition, CSCs in PDAC have been identified using cell surface markers, including CD133, CD44, CD24, and epithelial-specific antigen (ESA)/EpCAM [31]. It has been shown that the metastasis, chemoresistance, and relapse of PDAC are driven by CSCs. However, the molecular mechanisms responsible for the stemness of pancreatic CSCs remain unclear. A better understanding of the functions of these cells and the development of CSC inhibitors may contribute to tumor eradication [32–34].

The existence of CSCs in tumor tissues and the stem-like features of CSCs suggest that drugs targeting the stemness characteristics of CSCs may be used for cancer treatment. Tathiane et al. proposed the use of mRNAsi and EGER-mRNAsi to evaluate the stemness characteristics of CSCs [5]. Although the risk stratification of the stemness index has been investigated in pancancer populations, the comprehensive prognostic value of the stemness index for PADC is still unknown. Moreover, few studies have analyzed the genes related to the stemness characteristics of CSCs in PADC. Therefore, we aimed to develop a stemness-related prognostic signature for patients with PADC.

In this study, the K-M curves showed that there was a significant difference in the OS between the groups with low and high mRNAsi (mRNAsi/tumor purity) scores. The stemness index-related modules and genes were identified using WGCNA. The red module showed the highest positive correlation with the mRNAsi. A total of 36 genes related to the stemness of CSCs were obtained from this module. The GO, KEGG, and GSEA analyses showed that these genes were enriched in mitotic nuclear division, nuclear chromosome segregation, spindle checkpoint function, microtubule binding, and cell cycle. Next, a model comprising seven key mRNAsi-related genes (i.e., TPX2, ZWINT, UBE2C, CCNB2, CDK1, BUB1, and BIRC5) was constructed using univariate and multivariate Cox regression analyses. We found that the survival model accurately predicted the prognosis of PADC patients in both the TCGA and GEO databases. The ROC curves showed that this model has high predictive power for predicting the OS of PADC patients. Furthermore, some prognostic parameters (i.e., tumor size, risk score, and radiotherapy) were significantly correlated with the OS of PADC patients. Taken together, these results indicated that these genes might be related to the occurrence and development of PADC through regulating cell cycle, which were consistent with the study by Bai et al. [35]. Prospective studies are needed to verify the prognostic value of the stemness index-related signature in PADC.

Previous studies have reported that the dysregulation of the seven key genes contributes to the development of tumors [36–38]. In addition, these hub genes are closely associated with cell cycle events, such as mitotic cyclin destruction and cell cycle progression, and may be involved in carcinogenesis. *TPX2* encodes a microtubule-associated protein. The upregulation of TPX2 levels has been found to promote the proliferation and invasion of renal cancer cells [39]. ZWINT and CDK1, which correct erroneous centromere-microtubule attachment and regulate the mitotic spindle checkpoint, are mainly involved in cell cycle control in adrenocortical carcinoma [40]. The dysregulation of UBE2C is associated with the upregulation of Ki-67, a proliferative marker, and poor overall survival in colorectal carcinoma [41, 42]. Aaron et al. showed that aberrant expression of CCNB2 was closely related to cell cycle-driven subpopulation in advanced prostate cancer [43]. The *BIRC5* gene encodes survivin, an antiapoptotic protein that has been defined as a target in many cancers, including PDAC, and is overexpressed in PDAC [44, 45]. Few studies have investigated the genes related to the stemness characteristics of CSCs in PADC. Here, we showed that the seven genes regulated cell division cycle in PADC, suggesting that they may contribute to the initiation, metastasis, and recurrence of PADC. These findings support the development of therapies targeting the seven genes for PADC treatment. Future studies on the molecular mechanisms of these genes and the development of tailored targeted therapies are warranted.

## 5. Conclusion

In summary, we established a stemness index-related signature and developed a prognostic nomogram in combination with prognosis-related clinicopathological characteristics. This model might be used to predict the OS of patients with PADC. Furthermore, TPX2, ZWINT, UBE2C, CCNB2, CDK1, BUB1, and BIRC5 may orchestrate the stemness, proliferation, and invasion of tumor cells. These genes might be potential prognostic biomarkers and therapeutic targets in PADC.

## Data Availability

All data generated or analyzed during this study are included in the manuscript.

## Abbreviations

PADC: Pancreatic ductal adenocarcinoma
mRNAsi: mRNAsi expression base-index
EREG-mRNAsi: Epigenetic regulation based-index
DEGs: Differentially expressed genes
WGCNA: Weighted gene coexpression network analysis
CSCs: Cancer stem cells
FDR: False discovery rate
TOM: Topological overlap measure
GS: Gene significance
MS: Module significance
ME: Module eigengenes
MM: Module membership
GO: Gene Ontology analysis
KEGG: Kyoto Encyclopedia of Genes and Genomes Analyses
GSEA: Gene Set Enrichment Analysis
PPI: Protein-protein interaction
MF: Molecular function
BP: Biological process
CC: Cellular component
OS: Overall survival
ROC: Receiver operating characteristic curves
*C*-index: Concordance index.

## References

[1] Siegel R. L., Miller K. D., Jemal A. (2019). Cancer statistics, 2019. *CA: a Cancer Journal for Clinicians*.

[2] Rahib L., Smith B. D., Aizenberg R., Rosenzweig A. B., Fleshman J. M., Matrisian L. M. (2014). Projecting cancer incidence and deaths to 2030: the unexpected burden of thyroid, liver, and pancreas cancers in the United States. *Cancer Research*.

[3] Mizrahi J. D., Surana R., Valle J. W., Shroff R. T. (2020). Pancreatic cancer. *The Lancet*.

[4] Lee Y. T., Tan Y. J., Oon C. E. (2018). Molecular targeted therapy: treating cancer with specificity. *European Journal of Pharmacology*.

[5] Malta T. M., Sokolov A., Gentles A. J. (2019). Machine learning identifies stemness features associated with oncogenic dedifferentiation. *Cell*.

[6] Miranda A., Hamilton P. T., Zhang A. W. (2019). Cancer stemness, intratumoral heterogeneity, and immune response across cancers. *Proceedings of the National Academy of Sciences of the United States of America*.

[7] Valle S., Martin-Hijano L., Alcala S., Alonso-Nocelo M., Sainz B. (2018). The ever-evolving concept of the cancer stem cell in pancreatic cancer. *Cancers (Basel)*.

[8] Barabasi A. L., Gulbahce N., Loscalzo J. (2011). Network medicine: a network- based approach to human disease. *Nature Reviews. Genetics*.

[9] Li C., Heidt D. G., Dalerba P. (2007). Identification of pancreatic cancer stem cells. *Cancer Research*.

[10] Dalla Pozza E., Dando I., Biondani G. (2015). Pancreatic ductal adenocarcinoma cell lines display a plastic ability to bidirectionally convert into cancer stem cells. *International Journal of Oncology*.

[11] Di Carlo C., Brandi J., Cecconi D. (2018). Pancreatic cancer stem cells: perspectives on potential therapeutic approaches of pancreatic ductal adenocarcinoma. *World journal of stem cells*.

[12] Pan S., Zhan Y., Chen X., Wu B., Liu B. (2019). Identification of biomarkers for controlling cancer stem cell characteristics in bladder cancer by network analysis of transcriptome data stemness indices. *Frontiers in Oncology*.

[13] Pei G., Chen L., Zhang W. (2017). WGCNA application to proteomic and metabolomic data analysis. *Methods in Enzymology*.

[14] Langfelder P., Horvath S. (2008). WGCNA: an R package for weighted correlation network analysis. *BMC Bioinformatics*.

[15] Langfelder P., Zhang B., Horvath S. (2008). Defining clusters from a hierarchical cluster tree: the dynamic tree cut package for R. *Bioinformatics*.

[16] Niemira M., Collin F., Szalkowska A. (2019). Molecular signature of subtypes of non-small-cell lung cancer by large-scale transcriptional profiling: identification of key modules and genes by weighted gene co-expression network analysis (WGCNA). *Cancers (Basel)*.

[17] Wang C. C. N., Li C. Y., Cai J. H. (2019). Identification of prognostic candidate genes in breast cancer by integrated bioinformatic analysis. *Journal of clinical medicine*.

[18] Kanehisa M., Sato Y., Furumichi M., Morishima K., Tanabe M. (2019). New approach for understanding genome variations in KEGG. *Nucleic Acids Research*.

[19] Mazandu G. K., Chimusa E. R., Mulder N. J. (2017). Gene Ontology semantic similarity tools: survey on features and challenges for biological knowledge discovery. *Briefings in Bioinformatics*.

[20] Szklarczyk D., Morris J. H., Cook H. (2017). The STRING database in 2017: quality-controlled protein-protein association networks, made broadly accessible. *Nucleic Acids Research*.

[21] Blanche P., Dartigues J. F., Jacqmin-Gadda H. (2013). Estimating and comparing time-dependent areas under receiver operating characteristic curves for censored event times with competing risks. *Statistics in Medicine*.

[22] Zhang Z., Kattan M. W. (2017). Drawing nomograms with R: applications to categorical outcome and survival data. *Annals of translational medicine*.

[23] Subramanian A., Kuehn H., Gould J., Tamayo P., Mesirov J. P. (2007). *GSEA-P*: a desktop application for Gene Set Enrichment Analysis. *Bioinformatics*.

[24] Tochimoto M., Oguri Y., Hashimura M. (2020). S100A4/non-muscle myosin II signaling regulates epithelial-mesenchymal transition and stemness in uterine carcinosarcoma. *Laboratory Investigation*.

[25] Reya T., Morrison S. J., Clarke M. F., Weissman I. L. (2001). Stem cells, cancer, and cancer stem cells. *Nature*.

[26] Mani S. A., Guo W., Liao M. J. (2008). The epithelial-mesenchymal transition generates cells with properties of stem cells. *Cell*.

[27] Bonnet D., Dick J. E. (1997). Human acute myeloid leukemia is organized as a hierarchy that originates from a primitive hematopoietic cell. *Nature medicine*.

[28] Lapidot T., Sirard C., Vormoor J. (1994). A cell initiating human acute myeloid leukaemia after transplantation into SCID mice. *Nature*.

[29] Al-Hajj M., Wicha M. S., Benito-Hernandez A., Morrison S. J., Clarke M. F. (2003). Prospective identification of tumorigenic breast cancer cells. *Proceedings of the National Academy of Sciences*.

[30] Medema J. P. (2013). Cancer stem cells: the challenges ahead. *Nature Cell Biology*.

[31] Yang M. C., Wang H. C., Hou Y. C., Tung H. L., Chiu T. J., Shan Y. S. (2015). Blockade of autophagy reduces pancreatic cancer stem cell activity and potentiates the tumoricidal effect of gemcitabine. *Molecular Cancer*.

[32] Visvader J. E., Lindeman G. J. (2008). Cancer stem cells in solid tumours: accumulating evidence and unresolved questions. *Nature Reviews. Cancer*.

[33] Zhang M., Wang X., Chen X., Guo F., Hong J. (2020). Prognostic value of a stemness index-associated signature in primary lower-grade glioma. *Frontiers in Genetics*.

[34] Sancho P., Alcala S., Usachov V., Hermann P. C., Sainz B. (2016). The ever-changing landscape of pancreatic cancer stem cells. *Pancreatology*.

[35] Bai L., Ren Y., Cui T. (2020). Overexpression of *CDCA5*, *KIF4A*, *TPX2*, and *FOXM1* Coregulated Cell Cycle and Promoted Hepatocellular Carcinoma Development. *Journal of Computational Biology*.

[36] Wang F., Zhao W., Gao Y. (2019). CDK5-mediated phosphorylation and stabilization of TPX2 promotes hepatocellular tumorigenesis. *Journal of Experimental & Clinical Cancer Research*.

[37] Liu D., Xu X., Wen J. (2018). Integrated genome-wide analysis of gene expression and DNA copy number variations highlights stem cell-related pathways in small cell esophageal carcinoma. *Stem Cells International*.

[38] Song Y., Kim S., Lee H. (2020). Chromenopyrimidinone controls stemness and malignancy by suppressing CD133 expression in hepatocellular carcinoma. *Cancers (Basel)*.

[39] Liu B., Xiao Y., Li H. (2020). Identification and verification of biomarker in clear cell renal cell carcinoma via bioinformatics and neural network model. *BioMed Research International*.

[40] Xu W. H., Wu J., Wang J. (2019). Screening and identification of potential prognostic biomarkers in adrenocortical carcinoma. *Frontiers in Genetics*.

[41] Bavi P., Uddin S., Ahmed M. (2011). Bortezomib Stabilizes Mitotic Cyclins and Prevents Cell Cycle Progression via Inhibition of *UBE2C* in Colorectal Carcinoma. *The American Journal of Pathology*.

[42] Zhou Z., Cheng Y., Jiang Y. (2018). Ten hub genes associated with progression and prognosis of pancreatic carcinoma identified by co-expression analysis. *International Journal of Biological Sciences*.

[43] Horning A. M., Wang Y., Lin C. K. (2018). Single-cell RNA-seq reveals a subpopulation of prostate cancer cells with enhanced cell-cycle-related transcription and attenuated androgen response. *Cancer Research*.

[44] Liu S. H., Hong Y., Markowiak S. (2019). BIRC5 is a target for molecular imaging and detection of human pancreatic cancer. *Cancer Letters*.

[45] Huang X. Y., Qin W. T., Su Q. S. (2021). *Identification of a stemness-related prognostic model in pancreatic ductal adenocarcinoma by weighted gene co-expression network analysis*.

